# Mechanically induced single-molecule helicity switching of graphene-nanoribbon-fused helicene on Au(111)†

**DOI:** 10.1039/d1sc03976h

**Published:** 2021-09-14

**Authors:** Ayumu Ishii, Akitoshi Shiotari, Yoshiaki Sugimoto

**Affiliations:** Department of Advanced Materials Science, The University of Tokyo 5-1-5 Kashiwanoha 277-8561 Kashiwa Japan +81 4 7536 4058 +81 4 7536 3997; Department of Physical Chemistry, Fritz-Haber Institute of the Max-Planck Society Faradayweg 4-6 14195 Berlin Germany shiotari@fhi-berlin.mpg.de

## Abstract

Helicene is a functional material with chirality caused by its characteristic helical geometry. The inversion of its helicity by external stimuli is a challenging task in the advanced control of the molecular chirality. This study fabricated a novel helical molecule, specifically a pentahelicene-analogue twisted aromatic hydrocarbon fused with a graphene nanoribbon, *via* on-surface synthesis using multiple precursors. Noncontact atomic force microscopy imaging with high spatial resolution confirmed the helicity of the reaction products. The helicity was geometrically converted by pushing a CO-terminated tip into the twisted framework, which is the first demonstration of helicity switching at the single-molecule scale.

## Abstract 2

Graphene nanoribbon-fused helicene was fabricated *via* on-surface synthesis. The helicity of the product was reversibly switchable by pushing a CO-terminated scanning-probe-microscopy tip into the protruding helical terminal.

## Graphical abstract

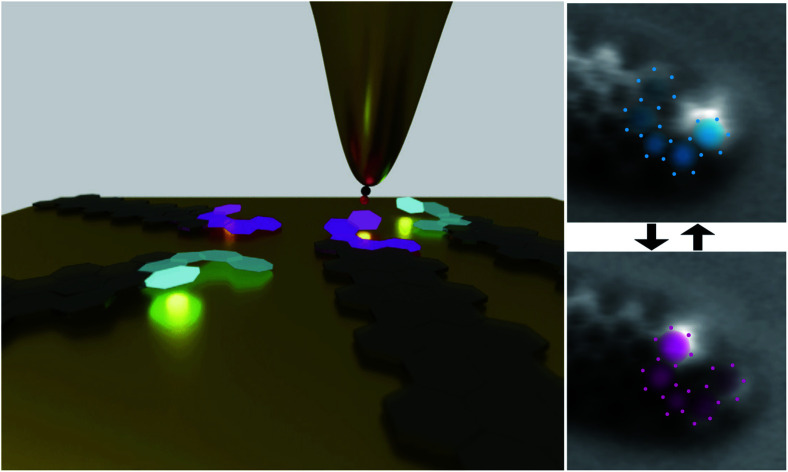

## 1 Introduction

Helicene, a nonplanar polycyclic aromatic hydrocarbon with a spiral shape, possesses chirality due to the structural difference between right- and left-handed winding (*i.e.*, helicity), which provides potential for application in molecular machines and devices.^1–3^ Scientific understanding of the unique properties of helicene has progressed to the single-molecule level; scanning probe microscopy (SPM), such as scanning tunnelling microscopy (STM) and atomic force microscopy (AFM), has enabled chiral discrimination^4–6^ and piezoelectricity measurements^7^ of individual helicene molecules on surfaces. Moreover, SPM has the potential to control the helicity of individual helicene molecules by applying a local stimulus from the probe tip. In the literature, single-molecule chirality control has been demonstrated using achiral molecules;^8–10^ these molecules are not chiral in the gas phase but acquire chirality by symmetry breaking upon adsorption to a surface.^11^ The voltage pulses from the STM tips to these molecules can induce configurational changes with the chirality inversion.^8–10^ However, the nanoscale manipulation of intrinsically chiral molecules has not yet been reported. The controllable and reversible inversion of helical structures, including helicene, by external stimuli is a challenge related to chemical, physical, and biological applications.^12,13^

**Scheme 1:**
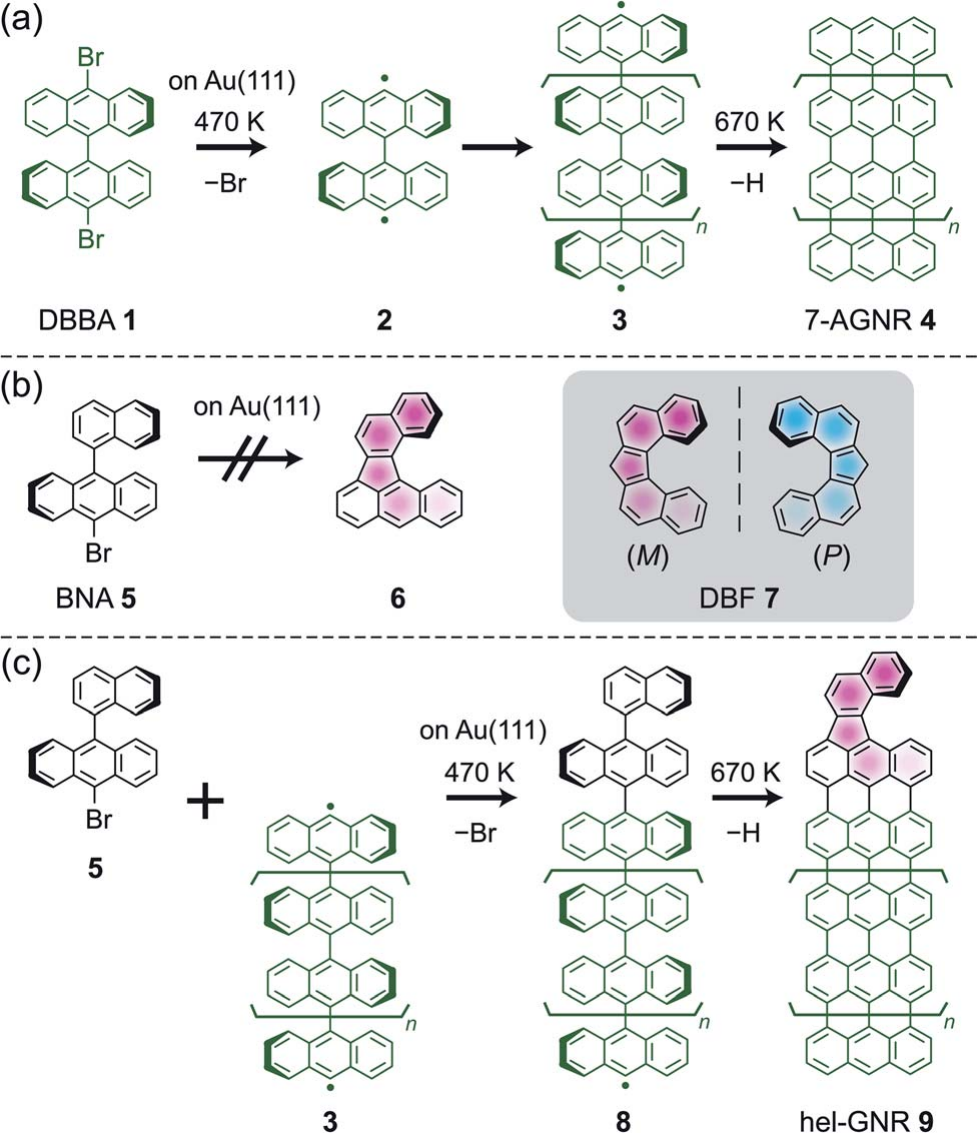
(a) Scheme for the fabrication of 7-AGNRs from DBBA on Au(111). (b) BNA precursor for DBF-type helicene. The helical product cannot be obtained from only the BNA precursor on Au(111). (c) Scheme for the fabrication of hel-GNRs from BNA and prepolymers on Au(111).

Helicity inversion has not yet been reported for the helicene molecules previously observed using SPM; thus, fabricating a novel helical compound is desirable for verifying the inversion ability. On-surface synthesis is a useful method for fabricating various aromatic compounds and nanocarbon materials, such as graphene nanoribbons (GNRs), with an atomically precise structure.^14–18^ As a key example, 10,10′-dibromo-9,9′-bianthracene (DBBA **1**) reacts on a Au(111) surface to afford armchair-edge GNRs with a width of seven carbon atoms (7-AGNRs **4**)^19^*via* debromination at ∼470 K (affording a diradical intermediate **2**), polymerisation by the radical C–C coupling (affording polyanthrylene intermediate **3**), and intrapolymer cyclodehydrogenation at 670 K (Scheme 1a). Using such a scheme, a wide variety of GNRs have been produced given the appropriate precursor design and substrate selection.^20–23^ Furthermore, on-surface synthesis can go beyond employing a single precursor species by using multiple precursors to create heterojunctions^19,24–28^ and terminal modifications,^29–32^ providing new functionality.

In this study, we fabricated GNRs with helicene-type terminals on a Au(111) surface using multiple precursors, identified the atomic structures of individual terminals using imaging at high spatial resolution, and controlled the helicity *via* local mechanical stimuli. During the on-surface reaction process, a potentially helical precursor was effectively fused with radical polymers to yield GNRs, which is a critical factor for obtaining the desired helical compound. The structures of the synthesised GNRs were identified using STM and AFM at 4.8 K. We demonstrated that the functionalised GNR terminal could be selectively and reversibly converted into its helical enantiomer by approaching the STM/AFM tip.

## 2 Results and discussion

We are convinced that a novel helical molecule would be produced from the cyclodehydrogenation of naphthylanthracene derivative **5** (Scheme 1b). The expected product **6** includes the dibenzo[*c*, *g*]fluorenyl (DBF **7**) framework. DBF is a pentahelicene analogue with a twisted V-shaped geometry (inset, Scheme 1b).^33,34^ Nevertheless, by annealing 9-bromo-10-(1-naphthyl)anthracene (BNA **5**) on a Au(111) surface, **6** was not obtained (Scheme 1b; Fig. S1 and the detailed description in ESI†) mainly because the small molecule tended to desorb from the surface at a high temperature.

The precursor must be strongly bound on the surface to invoke the targeted dehydrogenation reaction. Therefore, we used intermediate polymer **3** as an anchor to couple with BNA **5** (Scheme 1c) such that a larger intermediate **8** would undergo cyclodehydrogenation to afford GNRs with a helical terminal (hel-GNRs **9**). In other words, a radical of intermediate polymer **3** was capped by another precursor **5**. Subsequently, cyclodehydrogenation provided GNRs with functionalised terminals. This “radical capping” method is similar to the abovementioned heterojunction synthesis.^24^ However, the capping precursor **5** only had one halogen atom to enable the dehalogenated molecules to efficiently terminate radical polymers **3**, which would inhibit side reactions. For the sample preparation, we deposited DBBA **1** on a clean Au(111) surface and annealed it to yield intermediate polymer **3**, followed by BNA **5** deposition on the surface (see the detailed method in ESI†). Note that simultaneously depositing DBBA **1** and BNA **5** on clean Au(111) did not afford the desired product (Fig. S2 and detailed description in ESI†).

**Fig. 1:**
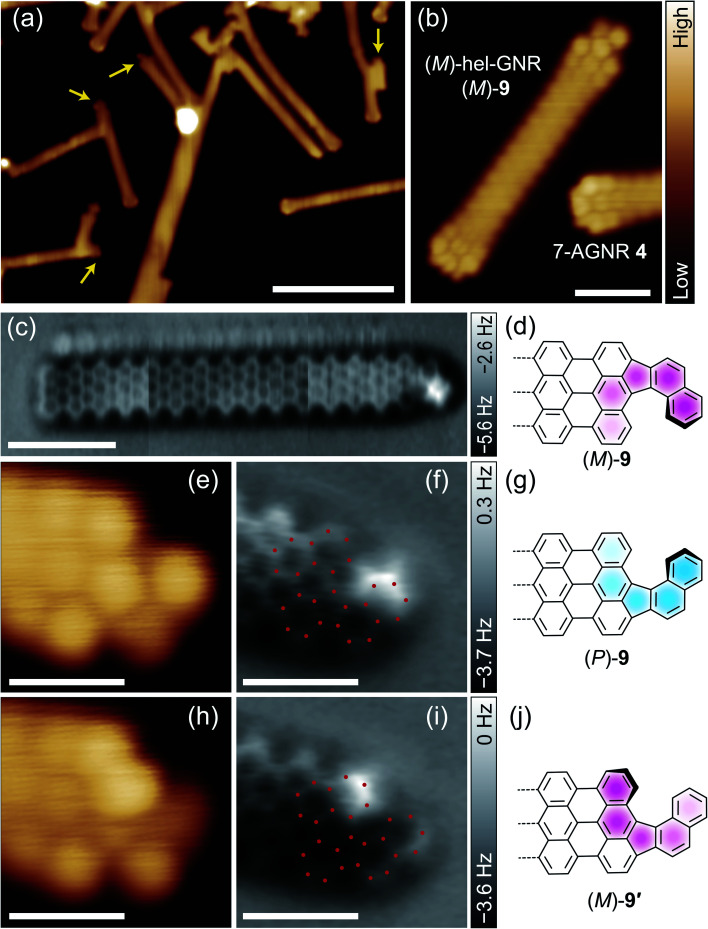
(a) Overview STM image of **3** + **5**/Au(111) after annealing at 670 K. (b) STM and (c) AFM images of an (*M*)-hel-GNR ((*M*)-**9**). The blobs at the upper part of the GNR are ascribed to Br atoms moved by the tip.^35^ The original AFM images are shown in Fig. S4.† (d) Structure of the right-side terminal of the GNR in (c). (e) STM image, (f) AFM image, and (g) structure of (*P*)-hel-GNR ((*P*)-**9**). (h) STM image, (i) AFM image, and (j) structure of (*M*)-hel-GNR in the alternate configuration ((*M*)-**9′**). As a guide to the eye, the red dots in (f) and (i) indicate the positions of the C atoms based on each image. The constant-current STM images were obtained with sample bias *V* = (a) 100 and (b, e, and h) 30 mV and tunnelling current *I* = 20 pA. The constant-height AFM images were obtained with *V* = 0 mV and tip height *z* of (c and f) 25 or (i) 50 pm. A positive (negative) *z* means that the height is higher (lower) than the STM set-point height over the Au surface (see ESI† for details). The scale bars represent (a) 10 nm, (b and c) 2 nm, or (e, f, h, and i) 1 nm.

Fig. 1a shows an STM image of the **3** + **5**/Au(111) sample after annealing at 670 K. GNRs with lengths of several tens of nanometers were observed. Some of their terminals were found to be capped by BNA-derivative structures (yellow arrows in Fig. 1a). A typical synthesised GNR is shown in Fig. 1b. The STM image of a typical 7-AGNR **4** terminal shows a symmetric nodal shape because of its localised electronic state near the Fermi level,^36^ whereas a BNA-derivative terminal is imaged as an asymmetric lobe (the upper part of Fig. 1b). The AFM image of the same ribbon resolved the positions of all C atoms (Fig. 1c), identifying the ribbon as (*M*)-hel-GNR (Fig. 1d). The helicity comes from the asymmetric terminal, *i.e.*, the DBF framework (see Scheme 1b and c). Notably, we did not find GNRs with both the terminals fused by helicene because (i) few GNRs have free terminals on both sides and many GNRs have a T-junction,^37^ a defective connection,^35^ or a terminal elongated from the surface step edges and (ii) the probability to cap both terminals is low owing to the relatively low deposition amount of BNA **5**. Furthermore, the DBF-type terminal was dominantly formed from the BNA precursor, and the side product of the cyclodehydrogenated BNA was not observed (Fig. S3 and the detailed description in ESI†).

In a constant-height AFM image of an adsorbate using a CO-terminated tip, a brighter spot corresponds to an atom protruding toward the vacuum, allowing helicity discrimination for individual adsorbates.^5,6^Fig. 1e and f shows STM and AFM images, respectively, of another GNR terminal. These images are mirror-symmetric to those shown in Fig. 1b and c, and therefore, this GNR was identified as (*P*)-hel-GNR (Fig. 1g). The helicity in the GNRs is attributed to the residual steric repulsion between the two adjacent hydrogen atoms at the terminal. In free space, hel-GNR **9** adopts only two geometries depending on its helicity. By contrast, each hel-GNR can assume either of the two nonequivalent configurations (*i.e.*, diastereomers) upon the adsorption onto the surface; either the naphtho side (Fig. 1b–g) or the anthra side (Fig. 1h–j) of the terminal protrudes toward the vacuum. The latter is a minor species that was rarely observed, probably because the main body (7-AGNR framework) tended to be flat because of large attractive interactions with the substrate. The AFM image of the minor configuration (Fig. 1i) indicates that only the edge of the anthra moiety protrudes toward the vacuum (Fig. 1j). Its helicity, *M*, is the same as that of the major species shown in Fig. 1b–d. To discriminate the two configurations of the same helicity, we here refer to the major and minor species as **9** and **9′**, respectively. Thus, the GNRs shown in Fig. 1b, e, and h were identified as (*M*)-**9**, (*P*)-**9**, and (*M*)-**9′**, respectively.

**Fig. 2:**
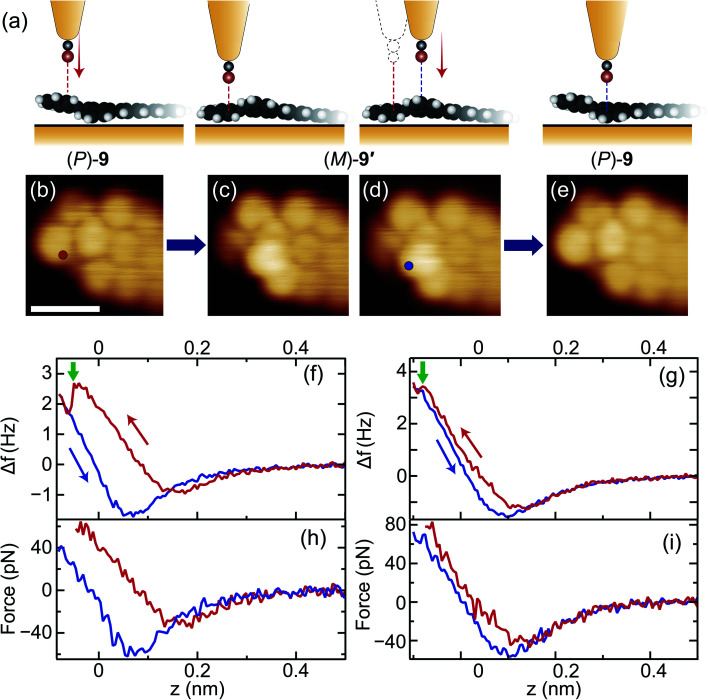
(a) Schematic side-view illustrations of reversible helicity switching of hel-GNR on Au(111) using a CO-terminated tip. The white, black, and red spheres represent H, C, and O atoms, respectively. The free space structure of hel-GNR was used for the illustrations. (b, c) STM images of a (*P*)-hel-GNR **9** (b) before and (c) after the approach of the tip to the protruding part of the GNR (red circle in (b); the left half of (a)). (d, e) STM images of a (*M*)-hel-GNR **9′** (d) before and (e) after the approach of the tip to the protruding part of the GNR (blue circle in (d); the right half of (a)). Images were obtained with *V* = 30 mV and *I* = 20 pA (scale bar: 1 nm). (f, g) Δ*f*(*z*) curves recorded during the tip approach (red) and retraction (blue) processes for “(b) → (c)” and “(d) → (e)” (*V* = 0 mV). The background curve recorded over the bare Au surface was subtracted (see ESI† in details). (h, i) Force curves calculated from the curves in (f) and (g).

We examined the switching ability of the synthesised hel-GNRs using a CO-terminated tip as illustrated in Fig. 2a. Fig. 2b shows an STM image of a hel-GNR, identified as (*P*)-**9** because of its shape match to the image in Fig. 1e. First, the lateral position of the tip was fixed over the protruding part of the naphtho moiety (red circle in Fig. 2b). Next, the frequency shift Δ*f* signals were recorded during the vertical approach of the tip to the sample (red curve in Fig. 2f). A sharp drop was observed at *z* = − 0.05 nm (green arrow in Fig. 2f), suggesting that a configuration change occurred at this tip height.^38,39^ After the event, the Δ*f*(*z*) curve during tip retraction (blue curve in Fig. 2f) exhibited a different shape from the onward curve; this difference also resulted from the configuration change. Finally, the STM image obtained after the Δ*f*(*z*) recording clearly indicates that the (*P*)-**9** was converted into (*M*)-**9′**. We note that no bias voltage was applied between the tip and sample during the tip approach-and-retraction process, and therefore the effects of tunnelling current can be excluded.

Backward conversion was also demonstrated using the same tip. We shifted the lateral tip position (see the third panel of Fig. 2a) to a fixed point over the protruding part of the converted GNR (*i.e.*, the edge of the anthra moiety of the terminal; blue circle in Fig. 2d) and then performed the Δ*f*(*z*) measurement (Fig. 2g). This process resulted in backward conversion: (*M*)-**9′** → (*P*)-**9** (Fig. 2e). We also confirmed the high reproducibility of tip-induced helicity conversion using other CO-terminated tips and other hel-GNR terminals. The conversion of (*M*)-**9** → (*P*)-**9′** was also induced (Fig. S7†), and reversible switching was achieved successively without destroying the tip or the sample (Fig. S5†).

Fig. 2h and i shows the force curves calculated from the curves in Fig. 2f and g, respectively, indicating that the conversion occurred when a vertical force of several tens of piconewtons was repulsively applied. This result was expected since CO-terminated tips are well known to cause local repulsive interactions with adsorbates due to the suppression of attractive forces from the metal tip body.^40,41^ We confirmed that metal-terminated tips providing large attractive forces did not induce the conversion of hel-GNRs (Fig. S8†). These results suggest that the “repulsive” CO-terminated tip pushed the protruding atoms of the hel-GNR toward the substrate and eventually flipped the twisted V-shaped framework. We have never observed the hel-GNR conversion other than during intentional tip-approach procedures, strongly indicating that the helical structure is substantially stable at low temperatures. We assume that the activation barrier for the helicity inversion of the hel-GNRs on the surface is comparable to that for DBF **7** ( ∼0.2 eV; see the detailed description in ESI†). Because the two configurations ((*P*)-**9** and (*M*)-**9′**) are inequivalent on the surface, the reaction barrier of (*P*)-**9** → (*M*)-**9′** should be different from that of the backward reaction ((*M*)-**9′** → (*P*)-**9**). However, we did not find a distinct difference in the ease of the tip-induced conversions, which implies that the barrier energy difference is small.

Notably, comparing Fig. 2f (h) and g (i), the shapes of the Δ*f* (force) curves are different. For example, the Δ*f*(*z*) drop during the first conversion (green arrow in Fig. 2f) was larger than that during the second (green arrow in Fig. 2g), and the difference between the force minima in the forward (red) and backward (blue) curves in Fig. 2f is larger than that in Fig. 2g. These features depended sensitively on the asymmetry of the tip apex and the subtle differences in lateral tip position (Fig. S6†). In all cases, however, it is common that (i) the force minimum of the backward curve after the conversion is smaller than that of the forward curve because of the indentation of the protruding part of the adsorbate and that (ii) the conversion occurred in the repulsion region.

To the best of our knowledge, this is the first study to control helicity at the single-molecule level. In most of the previous studies of helicene molecules adsorbed on surfaces, larger helical frameworks, such as heptahelicene, were targeted.^4,5^ The racemisation energies for such molecules are high (more than 1 eV); hence, no helicity conversion was observed in the previous STM studies. Furthermore, STM/AFM investigations of helicene molecules on metal surfaces have shown that tip approach dominantly causes lateral manipulation (inducing molecular diffusion on the surface) and vertical manipulation (picking the molecule up from the surface and attaching it to the tip apex).^5,42^ Such tip-induced movements are expected to be more pronounced for smaller molecules because of the weaker molecule–substrate interactions. Hence, to achieve tip-controlled helicity conversion, the target molecule should (i) retain steric geometry on the surface, resisting attractive adsorbate–substrate interactions; (ii) have an appropriate activation barrier; and (iii) be bound by a supporting molecule/material (fused GNR, in this case) to prevent the adsorbate from moving unintentionally. As described above, factor (iii) is also effective in evoking the desired cyclodehydrogenation in the on-surface synthesis scheme.

Not only from the viewpoint of the synthesis of a novel helical molecule, the reaction scheme in this study, namely, the “radical capping” method, is also noteworthy in terms of GNR functionalization. We expect that a variety of aryl monohalides can be used as capping precursors for the “radical capping” method; thus, a variety of complex and functional GNR terminals can be synthesised with this method. Furthermore, twisted and/or chiral GNRs are known to have characteristic mechanical, electronic, and magnetic properties.^43–45^ Exploring various types of helicene-fused GNRs and evaluating their applicable properties would be a promising subject for future research.

## 3 Conclusions

In summary, we fabricated helicene-terminated GNRs using the “radical capping” method. The capping precursor, namely BNA **5**, formed a DBF-derivative helical framework fused to 7-AGNRs on Au(111). The synthesised hel-GNRs exhibited the following four configurations: (*P*)-**9**, (*M*)-**9**, (*P*)-**9′**, and (*M*)-**9′**. The approach of the CO-terminated tip to the protruding part of the helical terminal caused a configurational change along with a helicity conversion: (*P*)-**9***⇌* (*M*)-**9′** and (*M*)-**9***⇌* (*P*)-**9′**. The twisted framework flip was driven by a local mechanical stimulus from the repulsive tip. Thus, we found a model system in which the helicity can be converted intentionally and reversibly at the single-molecule level. Such a selective conversion would greatly contribute to the fabrication of chirality/helicity-controllable molecular machines and devices.

## Data availability

The data that support the findings of this study are available from the corresponding author on reasonable request.

## Author contributions

Conceptualisation, methodology, software, and writing, A. S.; investigation, data curation, formal analysis, validation, and visualisation, A. I. and A. S.; funding acquisition, project administration, resources, and supervision, A. S. and Y. S.

## Conflicts of interest

There are no conflicts to declare.

## Supplementary Material

SC-012-D1SC03976H-s001
