# Supplementary material for: Mechanically induced single-molecule helicity switching of graphene-nanoribbon-fused helicene on Au(111)
Source: Chem Sci. 2021 Sep 14;12(40):13301–6. doi: 10.1039/d1sc03976h (PMC8528025; doi:10.1039/d1sc03976h)
Supplement: SC-012-D1SC03976H-s001 [file SC-012-D1SC03976H-s001.pdf]

Electronic Supplementary Material (ESI) for Chemical Science.
This journal is © The Royal Society of Chemistry 2021

Supporting Information

Experimental method

The experiments were conducted in an ultrahigh-vacuum chamber. A single-crystalline Au(111) surface was cleaned by Ar^+ sputtering and annealing at 770 K. DBBA **1** (Sigma-Aldrich) sublimated from an alumina crucible was deposited on the clean surface at room temperature. A deposition amount of approximately 30%–50% was suitable; smaller deposition amounts deteriorated the quality of 7-AGNRs,[1] whereas larger amounts made the remaining area for the adsorption of BNA **5** too small. The DBBA/Au(111) sample was annealed at 470 K for 10 min to form the polyanthrylene intermediate **3** (see Scheme 1a in the main text). Afterward, BNA **5** (Tokyo Chemical Industry) sublimated from another alumina crucible was deposited on the surface at room temperature. The deposition amount of BNA was confirmed by preliminarily observing a clean Au(111) surface after BNA deposition (Fig. S1a). The **3**+**5**/Au(111) sample was annealed at 470 K for 10 min and then at 670 K for 5 min to invoke cyclodehydrogenation. Subsequently, the sample was exposed to CO gas at 6 K so that a slight amount of CO molecules were adsorbed on the surface to fabricate CO-terminated tips.[2]

The in-situ microscopic measurement (Omicron low-temperature STM/AFM system) was conducted at 4.8 K. As a force sensor, a tuning fork with an etched W tip was used in frequency-modulation mode (resonance frequency: 21.3 kHz, spring constant: ∼1800 N/m, quality factor: 2–5 $\times 10^4$, and oscillation amplitude: 100 pm). All the

experimental images and curves in the main text were obtained using CO-terminated tips to enhance the spatial resolution.[2] For non-contact AFM images, frequency shift Δf was recorded in constant-height mode (sample bias $V = 0$ mV).[2] STM images were obtained in constant-current mode ($V = 30$ mV and tunnelling current $I = 20$ pA. The exceptions are the images shown in Fig. 1a and S1 as described in each caption). To obtain $\Delta f(z)$ curves, the lateral position of the tip was fixed, and then, the tip was approached to and retracted from the sample with $V = 0$ mV. Using the same tip, a $\Delta f(z)$ curve was also acquired over the bare Au surface (i.e., background curve). We confirmed that the background curve shows only a monotonic attractive contribution.[3,4] From the curves recorded over the molecules, the background curve was subtracted to remove the contribution of the Au surface. Force curves $F(z)$ were obtained from the $\Delta f(z)$ curves by the Sader formula.[5] The origin of the tip height z was the set-point height determined by STM over the bare surface ($V = 30$ mV and $I = 20$ pA). When $z < 0$, the tip is closer to the sample than the set-point height.

Optimisation of the synthesis procedure for helical products

Coupling with polyanthrylene intermediate **3** is an essential process for obtaining a helical product from BNA **5**. We confirmed that **5**/Au(111) without DBBA **1** does not react to afford helical products (Fig. S1f). Fig. S1a shows an STM image of **5** deposited on a clean Au(111) surface at room temperature without annealing. The molecules were clustered on the surface, forming one-dimensional zigzag chains, which is similar to the cluster formation of DBBA/Au(111)[1,6]. We assume that the bromoanthryl group of **5** lies on the surface, and the edge of the naphthyl group protruding toward the vacuum is captured as a protrusion in an STM image. After annealing at 520–570 K (Fig. S1b and c), the amount

of adsorbates decreased owing to the molecular desorption, and the residual molecules that were formed disordered GNRs. High-resolution AFM imaging (Fig. S1d) clarified that the cyclodehydrogenated BNA precursors were flattened and fused with each other via different carbon atoms from the radical site, forming heterogeneous structures. Such disordered GNRs were reported to be synthesised from DBBA/Ag(001)[7] and the chlorinated analogue of DBBA on Au(111)[8]. The possible reasons for helical products were not being synthesised (Fig. S1e) are as follows. (i) Single BNA molecules are expected to easily desorb from the surface at high temperatures (above 520 K). After annealing, most of the residual adsorbates are located near the step edges (Fig. S1b). (ii) When intermolecular cyclodehydrogenation occurs, the product becomes flattened and binds strongly to the Au(111) surface. These flattened hydrocarbon molecules tend to couple with each other in a disordered manner.[1,8] Therefore, to obtain the desired helical product, radical C–C coupling must properly occur before the intramolecular cyclodehydrogenation.

To confirm the importance of the two-step deposition process (Scheme 1c in the main text), we deposited both DBBA **1** and BNA **5** on a clean Au(111) surface at room temperature before annealing. Fig. S2 shows an STM image of the sample annealed at 670 K, indicating the formation of disordered GNRs like the BNA/Au(111) sample (Fig. S1b). We did not find hel-GNRs **9** on the surface (Fig. S2b). Almost all of the GNRs have defective structures,[1] which was probably because the presence of the coadsorbed BNA molecules inhibited the polymer formation by the Ullmann coupling between the DBBA molecules. For the sample shown in Fig. S2a, DBBA was deposited on clean Au(111) followed by BNA. Even when the deposition order was reversed to make a new sample, similar disordered GNRs were synthesised. Therefore, polyanthryl intermediate **3** should be prepared before the BNA **5** adsorption to invoke Ullmann coupling with the BNA-derived radical.

The cyclodehydrogenation for yielding hel-GNRs **9** from the **5**+**3**/Au(111) sample (Fig. S4a) is selective. Another possible reaction is shown in Fig. S4b, where another pair of sterically hindered hydrogen atoms was removed to form a new C–C bond, yielding a tetrahelicene-like structure (**10**). Although we obtained the images of >500 terminals of synthesised GNRs, **10** was not observed. According to the optimised structures in free space, for both cases, the twisting of the π-conjugated system remained due to the steric repulsion between two hydrogen atoms (Fig. S4c–e). The fjord region of **9** is larger than that of **10**; hence, **9** is expected to be more stable, which might contribute to the reaction selectivity. However, the interaction with the substrate can play a crucial role in the selectivity of on-surface synthesis products.[9,10] A more accurate evaluation would be provided by theoretical calculations of the adsorption structures and reaction pathways of the molecules on the surface.

Sequential switching

We conducted 11-time switching for an identical target using a CO-terminated tip. Fig. S5 shows the STM images obtained before and after the tip approaching processes. The images of the same configuration show the same appearance, indicating that the tip structure has not been changed by the sequential conversion. The spontaneous conversion was not observed unless the tip was manipulated to approach to the target, suggesting the stability of the configurations.

Fig. S6 shows some $\Delta f(z)$ curves recorded during this sequential switching operation. The curves in Fig. S6a and c (b and d) correspond to the conversions of (P)-**9** $\rightarrow$ (M)-**9′** ((M)-**9′** $\rightarrow$ (P)-**9**)). In both conversions, the clearness of the $\Delta f(z)$ jumps depends on the tip approach events. This suggests that the detected force varied depending on the slightest difference in the tip position. Because a CO molecule at the tip apex can

be tilted by repulsion with the atoms of the molecule on the surface, the vertical force detected with AFM varies greatly depending on the relative position of the tip to the target atom. Notably, in Fig. 2 of the main text, the detected repulsive force at the conversion (P)-**9** $\rightarrow$ (M)-**9′** is larger than at the backward conversion ((M)-**9′** $\rightarrow$ (P)-**9**)); however, in Fig. S6, the force values at the conversion (P)-**9** $\rightarrow$ (M)-**9′** are smaller. The variation in the detected force values can be attributed to the difference in asymmetry of CO at the tip apex, i.e., the relativity between the CO tilt direction and the orientation of the target molecule.

Verification of switching ability with different conditions

In the main text (Fig. 2), we displayed the conversion between (P)-**9** and (M)-**9′**. As shown in Fig. S7, its enantiomer, (M)-**9**, was also able to be switched by a CO-terminated tip located nearby. Fig. S7a and e shows STM images before and after the tip induction, respectively, indicating (M)-**9** $\rightarrow$ (P)-**9′**. Unlike the vertical tip approach process (Fig. 2 and S5), the conversion occurred when a constant-height AFM image (Fig. S7c) was obtained with a shorter tip height (Fig. S7b). The sudden change in the appearance (red arrow in Fig. S7c) indicates that the tip located just over the protruding region of the naphtho moiety induced the conversion, and the GNR was converted into (P)-**9′** after the point (Fig. S7d).

We also confirmed that metal-terminated tips were not able to induce the conversion between **9** and **9′**. A metal-terminated tip was approached to **9** and brought to the comparable distance in the case of CO-terminated tips (Fig. S8). At any tip positions, the repulsive force was not applied. Further tip approaching resulted in the destruction of the tip apex; this result suggests that the helicity conversion requires a repulsion force

from an approaching tip.

Estimation of the activation barrier for the helicity inversion

We recorded force curves during the helicity inversion (Fig. 2h and i). Nonetheless, the force relaxation of the tiltable CO molecule at the tip apex significantly modified the detected force.[11,12] Therefore, the force curves do not directly provide the exact barrier energy and threshold force values needed to activate the conversions.[4]

Here, we consider the activation barrier for helicity switching. For reference, pentahelicene, a helicene molecule consisting entirely of six-membered C rings, has an activation energy of 1.0 eV for racemisation.[13,14] Conversely, because of the broadened V-shaped angle of its molecular framework, the racemisation energy of DBF **7**, which includes a five-membered ring, is only 0.19 eV in free space.[14] This value is comparable with previously reported activation barriers of $\sim$0.2 eV for mechanically switchable single molecules on metal surfaces.[3,4] It is also known that intrinsically twisted molecules can be flattened by adsorption on a metal surface when the flipping barrier is very small, i.e., less than $\sim$0.1 eV.[10] In fact, GNRs with the tetrahelicene framework (cove-edged GNRs) adsorb flatly on the Au(111) surface;[15] a DFT calculation shows that the barrier of tetrahelicene is $\sim$0.15 eV.[16] Therefore, we assume that helicity flip for hel-GNRs on the surface has an activation barrier of approximately 0.2 eV similar to that for DBF **7**. For further verification, theoretical calculations of the adsorption structures would be needed.

References

[1] A. Ishii, A. Shiotari and Y. Sugimoto, *Nanoscale*, 2020, **12**, 6651–6657.

[2] L. Gross, F. Mohn, N. Moll, P. Liljeroth and G. Meyer, *Science*, 2009, **325**, 1110–1114.

[3] J. N. Ladenthin, T. Frederiksen, M. Persson, J. C. Sharp, S. Gawinkowski, J. Waluk and T. Kumagai, *Nat. Chem.*, 2016, **8**, 935–940.

[4] A. Shiotari, T. Odani and Y. Sugimoto, *Phys. Rev. Lett.*, 2018, **121**, 116101.

[5] J. E. Sader and S. P. Jarvis, *Appl. Phys. Lett.*, 2004, **84**, 1801–1803.

[6] G. Tian, Y. Shen, B. He, Z. Yu, F. Song, Y. Lu, P. Wang, Y. Gao and H. Huang, *Surf. Sci.*, 2017, **665**, 89–95.

[7] S. Smalley, M. Lahti, K. Pussi, V. Dhanak and J. Smerdon, *J. Chem. Phys.*, 2017, **146**, 184701.

[8] P. H. Jacobse, A. van den Hoogenband, M.-E. Moret, R. J. Klein Gebbink and I. Swart, *Angew. Chem. Int. Ed.*, 2016, **55**, 13052–13055.

[9] Q. Sun, L. Cai, Y. Ding, L. Xie, C. Zhang, Q. Tan and W. Xu, *Angew. Chem. Int. Ed.*, 2015, **54**, 4549–4552.

[10] A. Shiotari, T. Nakae, K. Iwata, S. Mori, T. Okujima, H. Uno, H. Sakaguchi and Y. Sugimoto, *Nat. Commun.*, 2017, **8**, 16089.

[11] Z. Sun, M. P. Boneschanscher, I. Swart, D. Vanmaekelbergh and P. Liljeroth, *Phys. Rev. Lett.*, 2011, **106**, 046104.

[12] A. J. Weymouth, T. Hofmann and F. J. Giessibl, *Science*, 2014, **343**, 1120–1122.

[13] R. Martin and M.-J. Marchant, *Tetrahedron*, 1974, **30**, 347–349.

[14] F. Pammer, Y. Sun, M. Pagels, D. Weismann, H. Sitzmann and W. R. Thiel, *Angew. Chem. Int. Ed.*, 2008, **47**, 3271–3274.

[15] J. Liu, B.-W. Li, Y.-Z. Tan, A. Giannakopoulos, C. Sanchez-Sanchez, D. Beljonne, P. Ruffieux, R. Fasel, X. Feng and K. Müllen, *J. Am. Chem. Soc.*, 2015, **137**, 6097–6103.

[16] S. Grimme and S. Peyerimhoff, *Chem. Phys.*, 1996, **204**, 411–417.

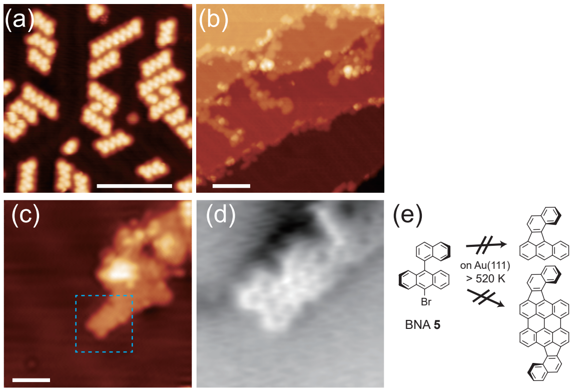

Figure S1: (a) STM image of BNA/Au(111) without DBBA adsorption (scale bar = 10 nm, V = 1 V, and I = 20 pA). (b) STM images of BNA/Au(111) after annealing at 570 K (scale bar = 10 nm, V = 300 mV, and I = 20 pA). (c) Magnified STM image of BNA/Au(111) after annealing at 520 K (scale bar = 2 nm, V = 30 mV, and I = 20 pA). (d) AFM image of the area surrounded by the blue square in (c) (z = 0 pm). (e) Reaction scheme of BNA **5** without DBBA **1**. The helical products similar to hel-GNRs were not observed for the BNA/Au(111) sample.

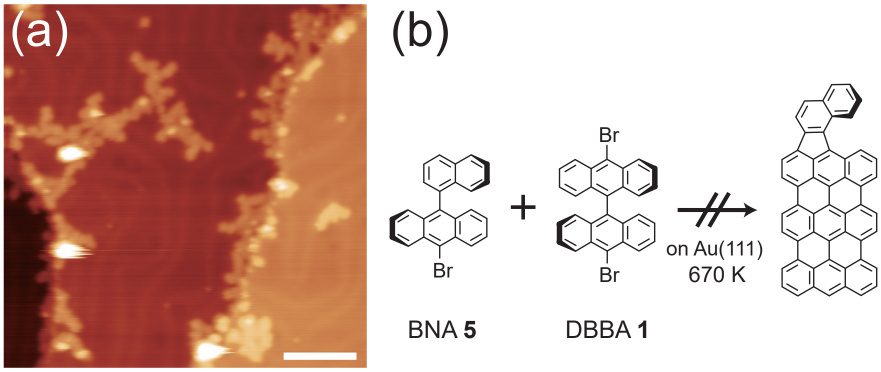

Figure S2: (a) STM image of Au(111) after the co-adsorption of DBBA **1** and BNA **5** at room temperature, followed by annealing at 670 K (scale bar = 10 nm, V = −300 mV, and I = 20 pA). (b) Reaction scheme of **1**+**5**/Au(111). The hel-GNRs were not obtained using this method.

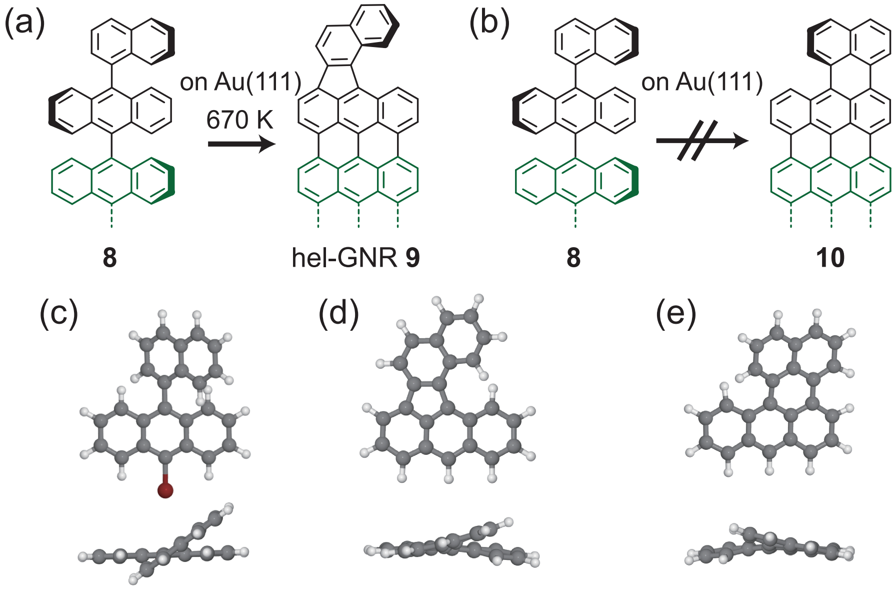

Figure S3: (a) Scheme of the cyclodehydrogenation of **8** for obtaining hel-GNR **9**. (b) Scheme for obtaining an isomer **10** that was not observed on Au(111). (c–e) Top (top) and side (bottom) views of the molecular structures of (c) BNA **5**, (d) naphthoanthracene similar to hel-GNR **9**, and (e) naphthoanthracene similar to **10**. The optimised structures were calculated in free space using the density functional theory (DFT)/B3LYP:3-21G* procedure.

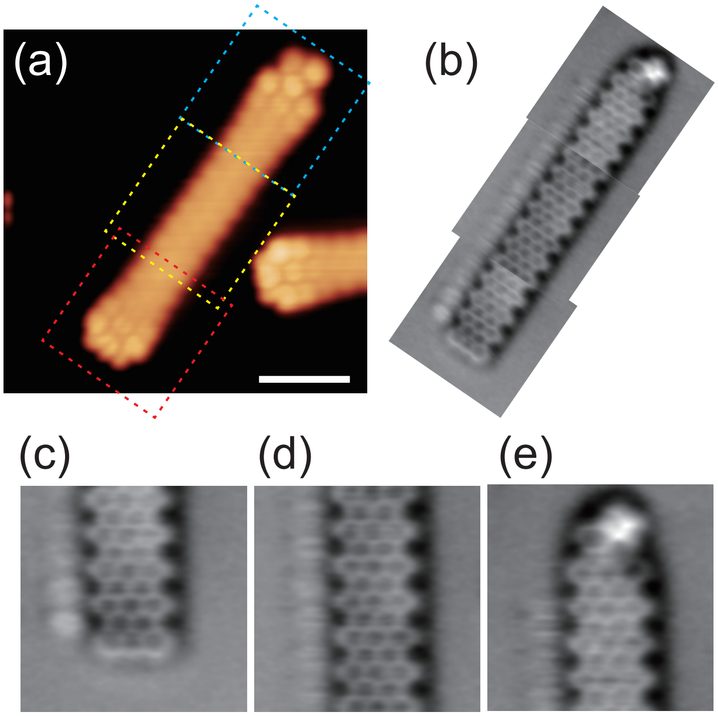

Figure S4: (a) STM image of (M)-**9** shown in Fig. 1b (scale bar = 2 nm). (b) AFM image of the GNR. (c–e) AFM images of the areas surrounded by the (c) red, (d) yellow, and (e) blue squares in (a). The three images were obtained by individual scans, and then combined and cropped to yield the overview image shown in (b) and Fig. 1c.

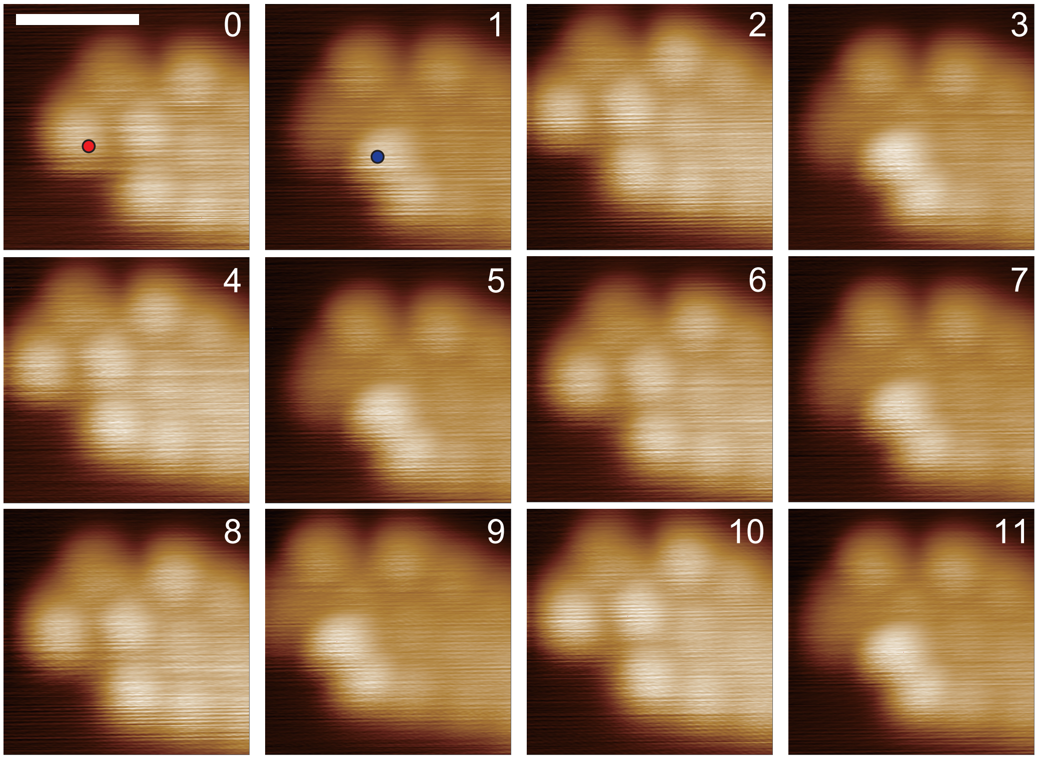

Figure S5: STM images of an identical hel-GNR on Au(111) at 4.8 K during sequential switching procedure using an identical CO-terminated tip. An even (odd) number tag in the upper right corner of each image represents (P)-**9** ((M)-**9′**). The point where the tip was approached to (P)-**9** ((M)-**9′**) is indicated by the red (blue) circle. Scale bar: 1 nm.

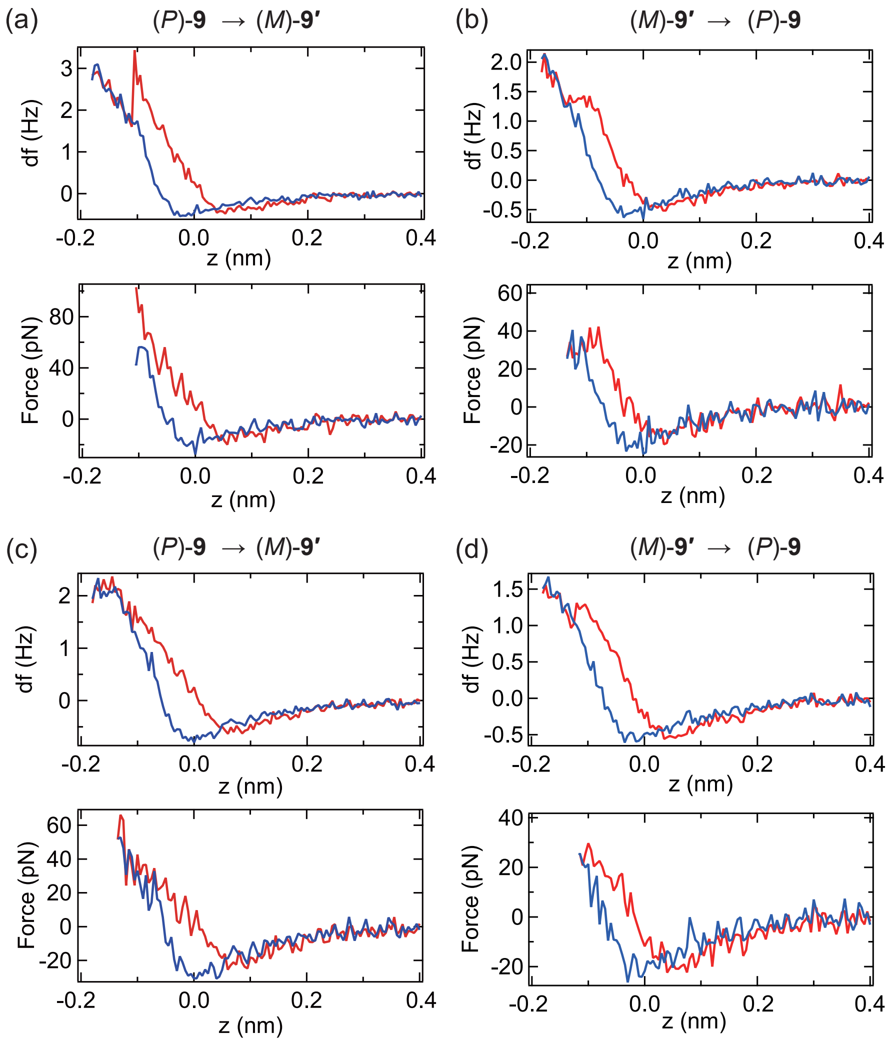

Figure S6: $\Delta f(z)$ (top) and $F(z)$ (bottom) curves for the tip approach (red) and retract (blue) process. The curves correspond to the conversion of (a) "6" → "7," (b) "7" → "8," (c) "8" → "9," and (d) "9" → "10" in Fig. S5.

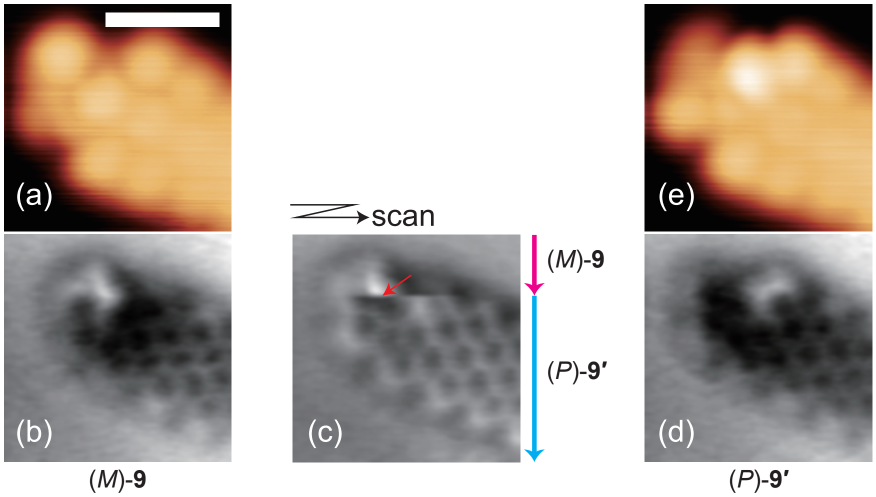

Figure S7: (a) STM and (b) AFM images of (M)-**9**. (c) AFM image of the GNR but with a shorter tip height. (d) AFM and (e) STM images of the GNR after scanning (c). The red arrow indicates the moment when the GNR was converted into (P)-**9′**. Scale bar: 1 nm. z = (b) −20, (c) −45, and (d) −35 pm.

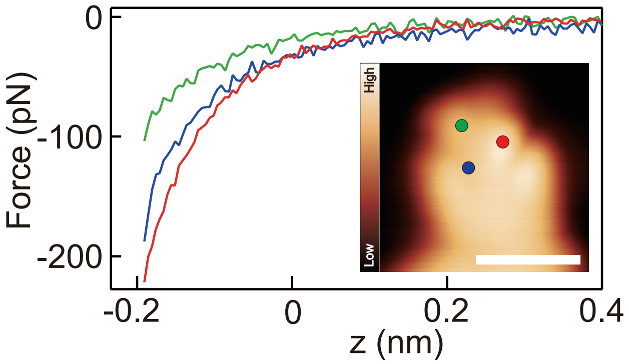

Figure S8: $F(z)$ curves for (M)-**9** using a metal-terminated tip. The inset shows an STM image of the target GNR (scale bar: 1 nm). The colours of the marker in the inset represent the tip positions of the recorded curves.
